# Sunflower Hybrid Breeding: From Markers to Genomic Selection

**DOI:** 10.3389/fpls.2017.02238

**Published:** 2018-01-17

**Authors:** Aleksandra Dimitrijevic, Renate Horn

**Affiliations:** ^1^Institute of Field and Vegetable Crops, Novi Sad, Serbia; ^2^Institut für Biowissenschaften, Abteilung Pflanzengenetik, Universität Rostock, Rostock, Germany

**Keywords:** association panel, genome-wide association studies, genomic estimated breeding value, genomic selection, genome sequence, marker-assisted selection, sunflower, traits

## Abstract

In sunflower, molecular markers for simple traits as, e.g., fertility restoration, high oleic acid content, herbicide tolerance or resistances to *Plasmopara halstedii, Puccinia helianthi*, or *Orobanche cumana* have been successfully used in marker-assisted breeding programs for years. However, agronomically important complex quantitative traits like yield, heterosis, drought tolerance, oil content or selection for disease resistance, e.g., against *Sclerotinia sclerotiorum* have been challenging and will require genome-wide approaches. Plant genetic resources for sunflower are being collected and conserved worldwide that represent valuable resources to study complex traits. Sunflower association panels provide the basis for genome-wide association studies, overcoming disadvantages of biparental populations. Advances in technologies and the availability of the sunflower genome sequence made novel approaches on the whole genome level possible. Genotype-by-sequencing, and whole genome sequencing based on next generation sequencing technologies facilitated the production of large amounts of SNP markers for high density maps as well as SNP arrays and allowed genome-wide association studies and genomic selection in sunflower. Genome wide or candidate gene based association studies have been performed for traits like branching, flowering time, resistance to *Sclerotinia* head and stalk rot. First steps in genomic selection with regard to hybrid performance and hybrid oil content have shown that genomic selection can successfully address complex quantitative traits in sunflower and will help to speed up sunflower breeding programs in the future. To make sunflower more competitive toward other oil crops higher levels of resistance against pathogens and better yield performance are required. In addition, optimizing plant architecture toward a more complex growth type for higher plant densities has the potential to considerably increase yields per hectare. Integrative approaches combining omic technologies (genomics, transcriptomics, proteomics, metabolomics and phenomics) using bioinformatic tools will facilitate the identification of target genes and markers for complex traits and will give a better insight into the mechanisms behind the traits.

## Introduction

Sunflower represents the second most important crop based on hybrid breeding, after maize (Seiler et al., 2017). It is mainly used for its seed oil, even though the seeds of confectionary sunflower also serve as snacks. With up to 12% of the global production of vegetable oils worldwide, sunflower takes position number four after palm oil, soybean and canola oil (Rauf et al., 2017). Apart from its use for human nutrition, sunflower oil has a number of industrial applications as, e.g., basic component for polymer synthesis, biofuel, emulsifier or lubricants (Dimitrijevic et al., 2017).

Up until the beginning of the 1970s of the last century sunflower production was based on open-pollinated varieties (Vear, 2016). Events that led to changing sunflower production to hybrid breeding were the discoveries of the first cytoplasmic male sterility (CMS) source (Leclercq, 1969) and the identification of corresponding restorer genes (Kinman, 1970; Leclercq, 1971). Soon after, in 1972, the first sunflower commercial hybrid was available for production in United States (Putt, 1978). Exploitation of heterosis for hybrid development enabled farmers to obtain higher seed and oil yields, as well as increased uniformity (Bohra et al., 2016). The development of sunflower hybrids set up sunflower as a major viable crop worldwide and encouraged the founding of numerous public and private breeding centers (Skoric, 2012; Seiler et al., 2017). In recent years, public and private sector contributed to assemble huge plant genetic resources in sunflower, to identify markers for marker assisted selection (MAS) and to establish the use of new high-throughput technologies in sunflower. Today, the estimated value of global sunflower production reaches $20 billion per year (FAO, 2016).

Basic directions in sunflower hybrid breeding include developing: (1) high seed and oil yield hybrids resistant to dominant diseases and tolerant to drought, (2) hybrids with changed oil properties, (3) confectionary hybrids, (4) herbicide resistant hybrids and (5) ornamental hybrids (Jocic et al., 2015). In addition, special markets have particular demands such as (1) achene and kernel properties as well as high protein content and lower oil content (lower than 40%) in confectionary sunflower production, (2) specific fatty acid and tocopherol composition in food and non-food industry or (3) plant height, ray and disk flower color, duration of flowering in ornamental sunflower hybrid breeding. The common needs for resistance against abiotic and biotic stress as well as the special needs of the various breeding purposes require the development of markers to facilitate the introduction of different traits.

**FIGURE 1:**
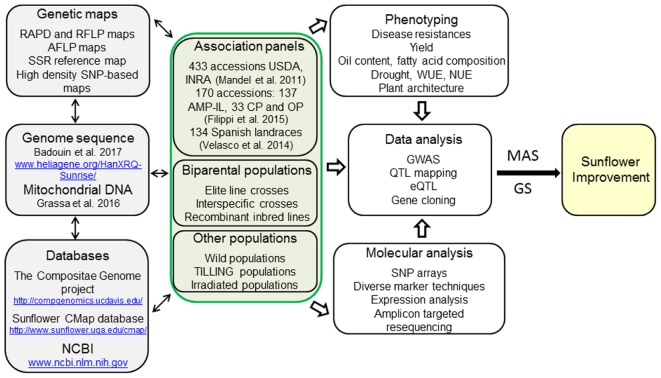
Schematic overview of the resources available in sunflower for marker-assisted selection (MAS) and future genomic selection (GS). Diverse plant genetic resources for sunflower breeding are available representing a large genetic diversity that can be exploited for sunflower improvement. The access to the sunflower genome sequences, the large resources of SNP, being part of high resolution maps or SNP arrays, and the huge amount of expression data will accelerate sunflower breeding by making the selection steps more efficient and precise. Future developments will move marker-assisted breeding toward genomic selection based on genomic estimated breeding values (GEBVs). WUE, water use efficiency; NUE, nitrogen use efficiency.

Botanically, sunflower (*Helianthus annuus* L.) is a member of the Asteraceae family, one of the most diverse and largest families of flowering plants. Due to the economic importance of the cultivated sunflower and the ecophysiological variability within the genus *Helianthus*, sunflower became a model plant species for genome studies in the family (Bachlava et al., 2012). The sunflower genome with 3.6 Gb is quite large (Badouin et al., 2017), three times larger than the rapeseed genome (Chalhoub et al., 2014), or more than eight times larger than the one of rice (Arumuganathan and Earle, 1991). Due to its ability to grow in different agroecological conditions and its moderate drought tolerance, sunflower may become the oil crop of preference in the future, especially in the light of global environmental changes. Even though simulations showed an increase of sunflower yield for northern parts of Europe in view of predicted climate changes, negative effects on sunflower yield may occur in southern latitudes (Debaeke et al., 2017). Consequently, more attention should be paid to breeding for better adaptation with regard to climate changes. These traits should include not only improvement in drought tolerance, but also introduction of pest resistance, salt tolerance and changes of plant architecture for better adaptation. Exploitation of available plant genetic resources in combination with the use of modern molecular tools for genome-wide association studies (GWAS) and application of genomic selection (GS) could lead to considerable improvements in sunflower. However, only in the recent years plant and genomic resources have become available in sunflower comparable to other crops (**Figure 1**). In this review we will talk about the long way that sunflower breeders and biotechnologists have to go and the future perspectives of using modern molecular tools in sunflower breeding.

## Plant Genetic Resources In Sunflower

### Biparental and Wild Populations

Biparental populations based on crosses between elite breeding, conventional, or introgressed lines (e.g., Berry et al., 1995; Horn et al., 2003; Vera-Ruiz et al., 2006; Kane et al., 2013; Livaja et al., 2016) as well as landraces and wild species (e.g., Quillet et al., 1995; Kim and Rieseberg, 1999; Brouillette et al., 2007; Ma et al., 2017) have been employed in sunflower for mapping of genes, marker detection, QTL analyses and gene cloning. In addition, recombinant inbred lines (RILs) have been developed that as immortals can be maintained forever by self-propagation (e.g., Berrios et al., 1999; Tang et al., 2002; Tang et al., 2006; Poormohammad Kiani et al., 2007a; Talukder et al., 2016). However, biparental populations have three major disadvantages: (1) these populations have to be individually established for each research project requiring time and resources, (2) only two alleles per locus can be evaluated and (3) due to missing recombination events populations show low resolutions in mapping (Bernardo, 2008; Patrick and Alfonso, 2013). The use of association panels overcomes these problems. To verify the usefulness of association panels the genetic diversity between wild populations and the genetic diversity fixed in association panels were compared (Mandel et al., 2011; Filippi et al., 2015). Even though alleles present only in the wild populations were detected, the majority of the alleles were present in the investigated association panels.

### Sunflower Collections

The largest sunflower collection is handled at the Institute of Field and Vegetable Crops, Novi Sad, Serbia consisting of over 7,000 sunflower inbred lines developed from different genetic sources and 21 perennial and 7 annual species (447 accessions in total)^1^ (Atlagic and Terzic, 2014). The next largest collection of more than 5000 cultivated and wild *Helianthus* accessions is held at the USDA-ARS NPGS in Ames (Marek, 2016). About half of these, 2,519 accessions, represent the world’s largest wild relatives sunflower collection, comprising 53 species – 39 perennial and 14 annual species (Seiler et al., 2017). Another large collection for sunflower (cultivated and wild) is maintained at the Vavilov Institute of Plant Industry, which consists of a total of 2,780 accessions from which 2,230 represent cultivated sunflower accessions and 550 wild sunflower accessions belonging to 24 species (19 perennials and 5 annuals) (Gavrilova et al., 2014). Some smaller numbers of 585 accessions of *H. annuus* are available via GRIN-CA, the Plant Gene Resources of Canada ^2^ and additional 613 sunflower accessions of diverse origin are distributed by the IPK Gatersleben ^3^. These resources represent mostly uncharacterized plant material. In contrast to these, a well-defined collection of 400 open-pollinated varieties, landraces and breeding pools has been assembled by INRA to reflect the worldwide diversity present in sunflower (Mangin et al., 2017b). However, conservation of population diversity of sunflower populations represents a challenge in the maintenance process due to the self-incompatibility of wild sunflowers (Gandhi et al., 2005) and the possibility of genetic drifts occurring during the propagation of seed stocks (Mangin et al., 2017b). To study the preservation of the genetic variability, a set of 114 cultivated sunflower populations of the INRA collection were genotyped using a 384 Golden Gate SNP Assay. In conclusion, multiplication in isolation fields or use of cages is recommended to reduce loss of genetic variability in cultivated genetic resources.

These worldwide available collections of sunflower represent a valuable resource for the sunflower community. It could be of interest to include some additional accessions of these large collections to the existing association panels described below.

### Association Panels

Association panels have to be characterized by molecular markers like SSRs or SNPs to avoid false associations due to the population structure and family relationship. The review here focusses on association panels that are online available as the prior mentioned sunflower collections. To analyze the primary gene pool of sunflower an association panel consisting of 433 cultivated accessions from North America and Europe in addition to 24 wild sunflower populations distributed over the whole of United States were characterized by 34 selected EST-SSRs chosen on the presumptive neutrality toward domestication and breeding efforts (Chapman et al., 2008; Mandel et al., 2011). USDA cultivated accessions in this panel were assigned to the following categories: HA and RHA being either non-oil or oil, landrace, open-pollinated variety (OPV), non-oil introgressed, oil introgressed, other non-oil, and other oil. The INRA accessions could only be categorized into INRA-HA and INRA-RHA as the information on oil and non-oil was not always available. Analyses using the software STRUCTURE (Pritchard et al., 2000) and Principle Coordinates (PCO) analyses (Patterson et al., 2006) did not reveal deep genetic divisions within the germplasm (Mandel et al., 2011). The cultivated and the wild populations separated into two different groups and within the cultivated accessions the restorer-oil (RHA-oil) category stayed apart from the remaining gene pool (Mandel et al., 2011). This is not unexpected due to the hybrid history of sunflower in which the maintainer and restorer pools have been kept separate on purpose to maximize heterosis (Fick and Miller, 1997). A selection of 288 accessions still covers nearly 90% of the genetic diversity available in the original larger panel. This association panel was named UGA-SAM1 and consists of 259 accessions, which are distributed by the Germplasm Resources Information Network (GRIN^4^) of the USDA National Plant Germplasm System (NPGS) and 29 accessions available through the French National Institute for Agricultural Research (INRA, France). This UGA-SAM1 population, which has been successfully employed in association studies (Mandel et al., 2013; Nambeesan et al., 2015), represents a very valuable tool for future association studies for the whole sunflower community. A minimal core set of 12 accessions (representing HA, RHA, oil and non-oil accessions as well as INRA material) capturing nearly 50% of the total allelic diversity might be ideal to build up a MAGIC (Multi-parent advanced generation intercross) population for sunflower. The MAGIC strategy is interesting for studies of multiple alleles in order to exploit higher recombination frequencies and better mapping resolution (Cavanagh et al., 2008). Development of MAGIC populations is in progress for numerous plant species (Bandillo et al., 2013) and would be interesting for sunflower as well.

Argentinean germplasm also represents a valuable genetic resource due to the long history of sunflower breeding in Argentina (de Bertero, 2003; Moreno et al., 2013). Filippi et al. (2015) characterized the population structure und calculated the genetic diversity of the association mapping population (AMP-IL), representing 137 INTA (National Institute of Agricultural Technology, Argentina) accessions and 33 accessions of open-pollinated and composite populations. The plant material is maintained by The Active Germplasm Bank of INTA Manfredi (AGB-IM). Using 42 SSR markers and/or SNP markers, detected by a 384 Illumina SNP-oligo pool array, estimated and observed heterozygosity as well as clustering using STRUCTURE and Discriminant Analysis of Principle Components (DAPC) were compared for the two marker types. As in other studies (Mandel et al., 2011, 2013) the population structure was dominated by the maintainer/restorer trait (Filippi et al., 2015).

A germplasm collection of 196 Spanish confectionary sunflower accessions is maintained at the Centre of Plant Genetic Resources of the National Institute for Agricultural and Food Research and Technology (CRF-INIA)^5^. A large genetic variation was revealed regarding hundred-seed weight, kernel percentage, seed oil content, fatty acid and tocopherol composition, phytosterols and other traits (Velasco et al., 2014; Pérez Vich et al., 2017).

In addition to well characterized association panels, considerable plant genetic resources are nowadays available in sunflower (cultivated as well as wild *H. annuus* accessions and accessions representing other species in the genus *Helianthus*).

### Mutagenized Populations

To increase the naturally available genetic variability sunflower has been mutagenized (Zambelli et al., 2015). Mutant populations have been successfully developed and used to screen for mutant phenotypes interesting for breeding purposes with regard to flowering time, dwarf habitus, oil content, high oleic trait, herbicide resistance and branching (Soldatov, 1976; Gabard and Huby, 2001; Sala et al., 2008a; Cvejic et al., 2011b; Leon et al., 2013). Recently, a TILLING (Targeted Induced Local Lesion In Genomes) population for high throughput screening of EMS (ethyl methane sulfonate)-induced mutations in sunflower was established by Sabetta et al. (2011) and used for studies of genes involved in the fatty acid biosynthesis. Optimized mutagenesis using EMS was used to develop an additional sunflower TILLING platform (Kumar et al., 2013). Phenotypic characterization of 5,000 M2 lines was performed to estimate the mutation rates and to select interesting mutants. As seed oil biosynthesis is of major importance in sunflower, TILLING of *FatA* and *SAD* genes were investigated and revealed an overall mutation rate of one mutation every 480 kb (Kumar et al., 2013). Another possibility to develop mutant populations is to apply gamma irradiation or fast neutrons (Cvejic et al., 2011a). Optimal ranges for gamma irradiation and fast neutrons were explored in comparison to EMS concentrations.

Besides induced mutagenized populations, natural mutations that have occurred in wild sunflower populations have had significant impact on sunflower hybrid breeding, especially in the area of herbicide resistance. In the recent years intensive use of herbicides has led to the emergence of resistant wild sunflower populations. The first case was a population of common sunflower found in a soybean field in Rossville (KS, United States), in which imazethapyr that belongs the group of AHAS (acetohydroxy acid synthase) inhibitors was used over a time course of seven consecutive years for weed control. Thus, creating the first sunflower population, named ANN-PUR, resistant to one of the AHAS inhibitors (Al-Khatib et al., 1998). Resistance from this population was successfully introduced into commercial sunflower hybrids (Miller and Al-Khatib, 2000; Jocić et al., 2004). Sunflower production based on the use of this imidazolinone (IMI) resistance, which provides an efficient and easy control of post-emergence broadleaf weeds in Europe, is called Clearfield^®^ technology. In addition to the discovery of IMI resistant sunflowers, another population of wild sunflowers (ANN-KAN), tolerant to another AHAS herbicide group called sulfonylurea, was discovered in Kansas (United States) (Al-Khatib et al., 1999). The same tolerance was also obtained by EMS mutagenesis (Gabard and Huby, 2001). Later, more populations of wild sunflowers resistant to AHAS herbicides were found (e.g., White et al., 2002, 2003; Jacob et al., 2017). In addition, a new tolerance for imidazoline called Clearfield Plus^®^ was selected from an M2 population of 600,000 plants treated with EMS (Sala et al., 2008a).

Natural genetic diversity and naturally occurring or chemically/gamma-ray induced genetic variability represent a perquisite for selection in breeding. The wide range of accessions maintained and made available by the germplasm banks for the research community is an extremely valuable starting point for successful breeding programs in sunflower allowing association studies and introduction of new traits into existing commercial breeding material. However, mutagenesis can create additional new genetic variability in traits where the natural variability is not sufficient.

## Genetic Maps and Sunflower Genome Sequence

Different molecular markers, which have been applied in mapping genes and development of sunflower linkage maps (**Figure 1**), set the basis for the assessment of the genetic diversity present in the genus *Helianthus* as well as in cultivated and wild sunflower accessions. Positioning of desirable genes allowed the identification and development of more specific molecular markers. At the Sunflower CMap database^6^ genetic maps available for sunflower have been listed and can be compared with each other by using the program CMAP (Kane et al., 2013).

The first map was developed on wild sunflower using RAPD markers (Rieseberg et al., 1993). A couple of years later maps were generated and published by using non-PCR based RFLP markers in different crosses of cultivated sunflower (Berry et al., 1995; Gentzbittel et al., 1995; Jan et al., 1998). These maps were published several years later than RLFP maps, e.g., in wheat, maize, barley, rice, and oilseed rape due to companies being involved in construction of the sunflower map (Hu, 2010). Later on, AFLP markers were added to the maps (Peerbolte and Peleman, 1996; Gedil et al., 2001). Most sunflower linkage maps contained 17 linkage groups (LG), representing the number of haploid chromosomes in sunflower. These maps were followed by genetic maps based on SSR markers (Tang et al., 2003b; Yu et al., 2003). The first composite genetic SSR map consisted of 278 single-locus SSR markers as well as additional 379 markers (public and proprietary), covering 1423 cM. This map that nowadays serves as reference genetic map for sunflower (Tang et al., 2003b) was then further saturated with additional SSR markers exploring three new mapping populations (Yu et al., 2003). In between more than 2,000 SSR have been derived from genomic sequences (gSSR) and EST (EST-SSR) and are now available for mapping and genotyping (Brunel, 1994; Dehmer and Friedt, 1998; Paniego et al., 2002; Tang et al., 2003b; Yu et al., 2003; Poormohammad Kiani et al., 2007b; Chapman et al., 2008; Heesacker et al., 2008). Existing sunflower maps were further enriched by these gSSRs, EST-SSRs, INDELs, TRAPs markers (Hu et al., 2007; Heesacker et al., 2008). These SSR markers (sequences and primers available through NCBI) represent a very valuable tool as they allow the localization of genes on individual linkage groups (Tang et al., 2003b) as well as on the recently published sunflower genome sequence of HanXRQ^7^ (Badouin et al., 2017). About 3 gigabases (Gb) representing 80% of the whole genome size were assembled and represent an extremely useful tool for all different research programs that aim at the improvement of sunflower hybrids.

Finally, the step toward high-density maps was made possible by using SNP-based markers, starting with Lai et al. (2005) who derived SNPs from an EST database (as part of the Compositae Genome Project) and used them for mapping. An Infinium Beadchip including 9,480 SNPs based on transcriptome data was developed by Bachlava et al. (2012) and employed by Bowers et al. (2012) to obtain four high-density genetic maps. Each of these maps contained 3,500–5,500 loci. Even though the maps were highly colinear, gaps in individual maps were observed. To solve this issue a consensus map of 10,080 loci was constructed from these data (Bowers et al., 2012). Talukder et al. (2014a) developed a high density map of 5,019 SNP markers obtained via RAD-sequencing. The rust resistance gene *R_12_* was fine-mapped using this SNP-based map. In addition, 118 SSR markers were included in the SNP map to address and orientate the linkage groups according to the sunflower reference genetic map. Celik et al. (2016) pioneered the use of genotyping-by-sequencing for large scale SNP detection in sunflower and developed a SNP-based linkage map of 817 SNP-markers covering all 17 LG by analyzing an F_2_ obtained from the cross RHA 436 × H08 M1. Using the newly developed 25 K SNP array in sunflower Livaja et al. (2016) were able to construct a linkage map based on 6,355 SNP markers for the RIL population NDBLOSsel × CM625. The connection between genetic linkage maps and the sunflower karyotype was finally made by developing a molecular cytogenetic map for *H. annuus* (Feng et al., 2013). BAC and BIBAC clones with known genetic locations were used in fluorescence *in situ* hybridization (FISH) experiments to address the individual chromosomes.

The high resolution of the recently developed high-density maps in sunflower facilitates to narrow down the regions of interest, which should allow identification and cloning of genes for various relevant traits in the near future. In addition, SNP-based maps deliver markers closely linked to, e.g., resistance genes that can be applied in large scale marker-assisted breeding programs or can be integrated in SNP arrays.

## Marker Development By Linkage Mapping

### Resistance to Downy Mildew

Developed linkage maps set a good basis for localization and mapping of simply inherited traits. Most of the downy mildew resistance genes, conferring resistance to the oomycete *Plasmopara halstedii*, have been found to be dominantly inherited and consequently, relatively easy to map by using molecular markers. Identification of closely linked markers also represents a good basis for map-based cloning of the genes.

Great efforts were put into examination of downy mildew resistance genes (*R* genes), designated as *Pl* genes, that are distributed throughout sunflower genome: *Pl_13_, Pl_14_, Pl_16_*, and *Pl_Arg_* on LG1; *Pl_1_, Pl_2_, Pl_6_, Pl_7_, Pl_15_*, and *Pl_20_* on LG8; *Pl_5_, Pl_8_*, and *Pl_21_* on LG13; *Pl_17_* and *Pl_19_* on LG4; while *Pl_18_* is localized on LG2 of the sunflower SSR reference map (Mouzeyar et al., 1995; Roeckel-Drevet et al., 1996; Vear et al., 1997; Bert et al., 2001; Slabaugh et al., 2003; Yu et al., 2003; Mulpuri et al., 2009; Wieckhorst et al., 2010; Bachlava et al., 2011; Vincourt et al., 2012; Qi et al., 2015a; Qi L.L. et al., 2016; Zhang et al., 2016; Ma et al., 2017). Most of *Pl* genes are clustered, except for *Pl_Arg_* and *Pl_18_*.

The *Pl* cluster on LG8 was the first to be detected by molecular markers. Mouzeyar et al. (1995) used RAPD and RFLP markers for mapping the first downy mildew resistance gene, *Pl_1_*, which is a part of a large *Pl* cluster (*Pl_1_, Pl_2_, Pl_6_, Pl_7_*). Of all genes in the cluster, *Pl_6_* gene was the most intensively examined since it conferred resistance to all the races present for a long time, except race 304. Different marker types were used for the introgression of *Pl_6_* into susceptible sunflower material including STS (Sequence-Tagged Sites) markers belonging to the TIR-NBS-LRR class of RGA (Resistance-Gene Analog) (Bouzidi et al., 2002) and co-dominant CAPS (Cleaved Amplified Polymorphic Sequence) markers (**Table 1**). The developed markers have been successfully used to introduce *Pl_6_* by MAS or to track the introduction of *Pl_6_* in backcrosses during conversion of downy mildew susceptible lines into resistant ones (Dimitrijevic et al., 2010; Jocic et al., 2010).

**Table 1:** Overview of resistance sources, locations, and markers of disease resistance genes in sunflower.

LG	Resistance	Source	Markers linked to the	Distance	Reference
	gene		resistance gene	(cM)	
LG1	*Pl_Arg_*	Arg1575-2^(1,2)^	NSA_007595^(4)^	0.01	Dußle et al., 2004^(1)^
		RHA 419^(3)^	NSA_001835^(4)^	0.01	Wieckhorst et al., 2010^(2)^
		RHA 464^(4)^	ORS 716^(2,3)^	0.3 ^(2)^; 0.0 ^(3)^	
			ORS 662^(1,2,3)^	1.9^(1)^; 0.3^(2)^; 0.0^(3)^	Imerovski et al., 2014b^(3)^
			ORS 675^(3)^	0.0	Qi et al., 2017^(4)^
			RGC52a, RGC52b, RGC151^(2)^	0.3	
	*Pl_13_*	HA-R5	ORS1008^(1)^	0.9^(1)^; 1.8^(2)^	Mulpuri et al., 2009^(1)^
			HT636^(2)^	2.2	Liu et al., 2012a^(2)^
	*Pl_14_*	29004	RGC203	1.6	Bachlava et al., 2011
			RGC188	2.6	
	*Pl_16_*	HA-R4	ORS1008	0.0	Liu et al., 2012a
			HT636	0.3	
LG2	*R_5_*	HA-R2	SFW03654^(2)^	0.6	Qi et al., 2012a^(1)^
			ORS653a^(1)^	1.8	Qi et al., 2015b^(2)^
			NSA_000267^(2)^	2.2	
			ORS1197-2^(1)^	3.3	
	*Pl_18_*	PI 494573	ORS203	0.4	Qi L.L. et al., 2016
			SFW03060	0.9	
			SFW03883	0.9	
			CRT214	1.1	
LG3	*Or_5_*	RPG01^(1)^	RTS05^(1)^	5.6	Lu et al., 2000^(1)^
		PHD^(2)^	CRT 392^(2)^	6.2	Tang et al., 2003a^(2)^
			ORS1036^(2)^	7.5	
LG4	*Pl_17_*	HA 458	ORS963	0.8	Qi et al., 2015a
			SFW04052	2.1	
	*Pl_19_*	PI 435414	NSA_003564	0.6	Zhang et al., 2017
			NSA_006089	0.6	
LG8	*Pl_1_*	RHA 266	S017H3-3	5.6	Mouzeyar et al., 1995
			S124EI-2	7.1	
	*Pl_6_*	HA 335^(1)^	Hap2R	0.0	Pankovic et al., 2007
		HA 336^(2)^	Hap3	0.0	
			CAPS HhaI	0.0	
			CAPS RsaI	0.0	
	*Pl_20_*	PI 494578	SFW02745/SFW09076/S8_ 11272025/S8_11272046	0.0	Ma et al., 2017
	*R_1_*	RHA 279	SCT06_950_	0.0	Lawson et al., 1998
LG11	*R_12_*	RHA 464	NSA_003426/NSA_004155/ NSA_000064^(2)^	0.83^(2)^	Gong et al., 2013a^(1)^
			CRT275^(2)^	1.0	Talukder et al., 2014b^(2)^
			ORS1227^(1)^	3.3	
			ZVG53^(1)^	9.6	
	*R_14_*	PH3	NSA_000064	0.7	Zhang et al., 2016
			ORS1227	1.6	
			ORS542	3.5	
			ZVG53	6.9	
LG13	*Pl_5_*	XRQ	Ha-NT5R1,	4.8	Radwan et al., 2004
			Ha-NT5S3	6.4	
			Ha-NT5S1/Ha-NT5S2/ Ha-NT5R2	13.6	
	*Pl_8_*	QIR8 (*Pl_8_*	RGC251^(2)^	0.3	Radwan et al., 2004^(1)^
		derived from RHA 340)^(1)^	RGC15^(2)^	0.4	Bachlava et al., 2011^(2)^
		RHA 340^(2,3)^	NSA_000423/SFW01497/ SFW08875^(3)^	0.4	Qi et al., 2017^(3)^
			Ha-NT8R3/Ha-NT8R4^(1)^	3.5	
			Ha-NT8R5/Ha-NT8R6^(1)^	4.8	
			Ha-NTIR11gEI^(1)^	6.3	
	*R_4_*	HA-R3^(1,2)^	SFW05240/SFW05630/SFW06095/ SFW08283^(2)^	0.6	Qi et al., 2011b^(1)^
			SFW01497/SFW05630/SFW08875^(2)^	0.7	Qi et al., 2015b^(2)^
			ORS581^(1)^	0.8	
			ZVG61^(1)^	2.1	
	*R_HAR6_*	HA-R6	ZVG61	0.7	Bulos et al., 2013a
			ORS581	1.5	
	*R_13a_*	HA-R6	SFW05743^(2)^	0.2	Gong et al., 2013b^(1)^
			RGC15/16^(1)^	0.3	Qi et al., 2015b^(2)^
			ORS316/ZVG61^(1)^	0.4	
			ZVG61^(2)^	0.4	
			SUN14^(1)^	0.8	
	*R_13b_*	RHA 397	SUN14^(1)^	0.0	Gong et al., 2013b^(1)^
			SFW00757^(2)^	0.0	Qi et al., 2015b^(2)^
			RGC15/16^(1)^	0.1	
			ZVG61, ORS316^(1)^	0.3	
			SFW04275/SFW04317/SFW05743^(2)^	2.4	
			ZVG61, ORS316^(2)^	5.9	
	*R_adv_*	‘Advance’^(1)^	SCX20_600_^(1)^	0.0	Lawson et al., 1998^(1)^
		RHA 340^(2)^	RGC260^(2)^	0.2	Bachlava et al., 2011^(2)^
			ORS316^(2)^	3.0	
	*P_u6_*	P386	ORS316	2.5	Bulos et al., 2014
			ORS224	4.8	
	*R_11_*	Rf ANN-1742	ORS728	0.3	Qi et al., 2012b
			ORS45	1.0	
	*Or_ab-vl-8_*	AB-VL-8	ORS683	1.5	Imerovski et al., 2016
			ORS657	4.7	
LG14	*R_2_*	MC 29 (USDA)	SFW01272	1.8	Qi et al., 2015c
			NSA_002316	2.8	
			SFW00211	2.9	

*Numbers are brackets in the superscript in columns: “Source” and “Markers linked to the resistance gene” refer to the author citation list superscript numbers given in the “Reference” column concerning a specific resistance gene.*

Two *Pl* genes originating from *H. argophyllus, Pl_8_* and *Pl_Arg_*, were also subject of numerous studies. *Pl_Arg_* confers resistance to all present downy mildew races and *Pl_8_* to 96% of all isolates collected in the north-central region of United States (Gilley et al., 2016). Radwan et al. (2004) developed STS markers for detection of the *Pl_5_*/*Pl_8_* locus that were later on also explored in other sunflower genotypes (Dimitrijevic et al., 2011). Bachlava et al. (2011) developed two resistance gene candidate (RGC) markers, RGC251 and RGC15/16, closely linked to *Pl_8_* that belong to the group of SSCP (Single-Strand Conformational Polymorphism) markers. However, SSCPs are labor-intensive and time-consuming in MAS. A comprehensive study of *Pl_8_* by Qi et al. (2017) explored previously published SNP markers as well as two SSR markers (Bowers et al., 2012; Talukder et al., 2014a) to genotype a F_2_ population derived from the cross HA434 × RHA340. The three closest SNP markers, NSA_000423, NSA_002220, and NSA_002251, were then investigated to check the specificity of the identified markers, concluding that NSA_000423 and NSA_002220 could serve as diagnostic markers in 87% of the tested sunflower lines when RHA 340 is used as donor for the *Pl_8_* gene. Validation of these markers across 548 sunflower lines proved their usefulness for MAS. However, a larger panel of sunflower lines should to be tested.

Unlike, the *Pl_8_* gene, *Pl_Arg_* is not clustered. Several authors identified and developed different types of markers (SSRs, SNPs, RGCs) for MAS (Dußle et al., 2004; Wieckhorst et al., 2010; Imerovski et al., 2014b), some of these were also validated across a panel of sunflower lines. ORS716 was identified as the most useful marker in MAS (**Table 1**). Recently, Qi et al. (2017) combined available genomic data for the population obtained from the cross HA 89 × RHA 464 by use of SNP markers (Pegadaraju et al., 2013; Talukder et al., 2014a) with the phenotypic evaluation for resistance. The two nearest SNP markers (NSA_007595 and NSA_001835) narrowed the *Pl_Arg_* locus down to an area of 2.83 Mb. The nine identified SNP markers represent valuable diagnostic tools for introgression of *Pl_Arg_* into most genetic backgrounds in sunflower.

Other markers for use in MAS for downy mildew resistance include the identification of the tightly linked SSR marker ORS1008 to *Pl_13_* gene (Mulpuri et al., 2009), development of RGC markers tightly linked to *Pl_14_* gene (Bachlava et al., 2011) and the identification of one dominant co-segregating SSR marker (ORS1008) and one co-dominant tightly linked (EST)-SSR (HT636) to *Pl_16_*. Interestingly, HT636 and ORS1008 were reported to be linked to both, *Pl_13_* and *Pl_16_*, indicating that these genes are in close vicinity to each other (Liu et al., 2012a). Qi et al. (2015a) used SSRs to place *Pl_17_* onto LG4 and then used SNPs identified by the National Sunflower SNP Consortium (Talukder et al., 2014a) and by Bowers et al. (2012) to saturate the region surrounding the *Pl_17_* gene. The authors identified SNP SFW04052 and ORS963 as the closest flanking markers linked to *Pl_17_*. A year later, Qi L.L. et al. (2016) used the same methodology to map *Pl_18_* to LG2 and found two SSRs and 10 SNPs flanking the *Pl_18_* gene. *Pl_18_* represents the first gene mapped to LG2. In 2017, two new *Pl* genes, *Pl_19_* and *Pl_20_*, were reported and mapped to LG4 and LG8, respectively (Ma et al., 2017; Zhang et al., 2017). Two SSRs and two SNPs were mapped in close vicinity to *Pl_19_*, while four SNP markers (SFW02745, SFW09076, S8_11272025, and S8_11272046) co-segregated with *Pl_20_*. All markers can be used in MAS and most importantly in pyramiding *Pl* genes in order to achieve long lasting resistance toward downy mildew. The development of SNP markers is of special interest because of the large number of markers generated that increase the likelihood to have markers available for any cross combination.

### Resistance to Sunflower Rust

Infections of sunflower plants with *Puccinia helianthi* Schwein lead to the rust disease. This fungus, which is mostly spread in North America, Argentina, South Africa, and Australia, can cause significant damage and yield reduction in infected fields. Genetic control of the disease can be effective; however, due to fast emergence of new races either by sexual or asexual reproduction, resistance achieved is short-termed. Consequently, a significant effort has been made into discovering rust resistance genes and the introduction into commercial lines and hybrids with a final goal of pyramiding several resistance genes in order to achieve long-term resistance. Most of the rust resistance genes (*R* genes), described so far, are monogenic dominant. *R* genes are located on different LGs of the sunflower genome with the majority being located on the LG13 [*R_4_, R_u6_, R_11_, R_adv_, R_13a_* (*R_HAR6_*), and *R_13b_*] (Bachlava et al., 2011; Qi et al., 2011b, 2012b; Gong et al., 2013b; Bulos et al., 2014).

First molecular studies were conducted on discovering markers for *R_1_* and *R_adv_* genes by use of RAPD and SCAR markers (Lawson et al., 1996, 1998). While *R_1_* gene was the first rust resistance gene present in a large number of sunflower lines, *R_adv_* is present in the line P2 owned by Pioneer Hi-Bred Australia (Lawson et al., 1998; Qi et al., 2011a). *R_adv_* is also present in the USDA line RHA 340, which Bachlava et al. (2011) used for mapping of the gene. Lawson et al. (1998) developed the SCAR marker SCT06_950_ linked to *R_1_* gene, which proved to be useful for detection of *R_1_* in different genetic backgrounds, except for the sunflower line MC29, which carries the *R_2_* and *R_10_* genes. For mapping of *R_2_*, Qi et al. (2015c) used a different MC29 line, called MC29 (USDA) as it was cultivated in the USDA-ARS Sunflower Research Unit, Fargo, North Dakota, which differs in term of resistance to NA race 6 in comparison to the MC29 line used by Lawson et al. (1998). Qi et al. (2015c) reported two SNP markers, NSA_002316 and SFW01272, flanking the *R_2_* gene on LG14. Since, the closest marker, SFW01272, can only to a certain extent be used to detect the *R_2_* gene across different genetic backgrounds; the authors recommend the use of two flanking SNP markers in order to minimize selection of false positives in MAS.

Further molecular studies of *R* genes include identification of molecular markers closely linked to *R_4_, R_adv_, P_u6_, R_11_, R_13a_* (*R_HAR6_*), and *R_13b_* genes that are located on LG13. Qi et al. (2011b) identified two markers flanking *R_4_* gene (ORS581 and ZVG61) in the cross HA 89 × HA-R3, which were later also reported to be linked to rust resistance genes *R_13a_* (*R_HAR6_*) and *R_13b_* located on the lower end of the LG13 (Bulos et al., 2013a; Gong et al., 2013b; Qi et al., 2015b) (**Table 1**). Further on, Gong et al. (2013b) saturated the region flanking the genes by analysis of RGC markers that were present in vicinity of downy mildew resistance gene *Pl_8_*, which was also mapped in the lower end of LG13. Another *R* gene that mapped in vicinity of *Pl_8_* and fertility restorer gene *Rf_1_* was *R_adv_*. A completely co-segregating SCAR marker (Lawson et al., 1998) as well as RGC and SSR markers tightly linked to *R_adv_* were identified (Bachlava et al., 2011) (**Table 1**). Recently, Bulos et al. (2014) mapped *P_u6_* gene and identified closely linked SSRs to this gene in the sunflower line P386 on lower end of LG13. However, these markers are too far away to be useful in MAS (**Table 1**). *P_u6_* and *R_4_* map 6.25 cM apart from each other. Qi et al. (2012b) examined the *R_11_* gene and mapped it 1.6 cM from fertility restoration gene *Rf_5_* also on the lower end of LG13, hypothesizing the presence of a great rust R-gene cluster of *R_adv_*/*R_11_*/*R_4_*. SSR marker ORS45 was the closest to *R_11_* gene and was mapped 1 cM proximal to the gene, while ORS728 was shown to be a common marker for *R_11_* and *Rf_5_* genes. The results allow the conclusion that the lower end of LG13 harbors the second largest cluster of NBS-LRR encoding genes: rust resistance and downy mildew resistance genes. Based on SSR and RGC markers used in this area, Gong et al. (2013b) proposed that this big cluster could be sub-divided into two clusters. *R_adv_* and *R_11_* form sub-cluster I, while *R_4_, R_13a/b_, Pl_5_, Pl_8_* form subcluster II. *Pl_21_* that was also positioned on LG13 mapped 8 cM proximal to *Pl_5_*/*Pl_8_* (Radwan et al., 2004; Vincourt et al., 2012).

Other rust resistance genes investigated by use of molecular markers include analysis of *R_5_*. This is to date the only *R* gene discovered on LG2. Qi et al. (2012a, 2015b) identified two SSR and two SNP markers flanking the gene, with the closest being 0.6 cM away (**Table 1**). On LG11 two rust resistance genes have been mapped so far: *R_12_* and *R_14_*. Both genes were positioned in the middle of LG11, and were discovered in wild sunflower accessions, however, they have different origin: *R_12_* from PI413047 and *R_14_* from PI413038 (Gong et al., 2013a; Zhang et al., 2016). Both genes were mapped between the markers ORS1227 and ZVG53 (ORS1227 with 3.3 and 1.6 cM and ZVG53 with 9.6 and 6.9 cM from *R_12_* and *R_14_*, respectively). Talukder et al. (2014b) performed fine mapping of the *R_12_* gene region by using SNP markers. Five SNP markers (NSA_000064, NSA_008884, NSA_004155, NSA_003320, and NSA_003426) were linked with 0.83 cM to the gene, but only two markers (NSA_003426 and NSA_004155) proved to have diagnostic quality for *R_12_* (**Table 1**). The nearest SNP marker to *R_14_* was NSA_000064, which was mapped with 0.7 cM from the gene in the F_2_ mapping population obtained from the cross HA 343 × PH3 (Zhang et al., 2016). However, this marker amplified the same banding pattern in RHA 464 (*R_12_*) and PH3 (*R_14_*). Zhang et al. (2016) identified thirteen SSR/InDel and two SNP markers that amplified different profiles between the two donors of *R_12_* and *R_14_* indicating polymorphisms between these regions.

One of the latest efforts in saturation mapping of *R* genes was published by Qi et al. (2015b) who used previously developed SFW and NSA SNP markers in order to saturate the regions surrounding *R_4_, R_5_, R_13a_*, and *R_13b_* genes and succeeded in identifying markers that are under 1 cM distant from all analyzed genes thus raising the efficiency of introduction of rust resistance in to susceptible material (**Table 1**). The authors used previously developed SSR markers and newly developed SNP markers for identification of homozygous “double-resistant” F_2_ individuals in a population obtained from a cross combination between a BC_3_F_2_ plant harboring *R_5_* and HA-R6 bearing *R_13a_*. The F_4_ progeny obtained from chosen plants showed improved resistance toward races 336 and 777 in comparison to lines that possess only one resistance gene. Qi et al. (2015c) also performed marker-assisted pyramiding of *R_2_* and *R_13a_* in confectionary sunflower by use of SSR and SNP markers. Further pyramiding of *R* genes could lead to long-term improvements in sunflower rust resistance. The process of converting susceptible into resistant forms can be greatly facilitated and accelerated by use of the reported molecular markers.

### Resistance to Broomrape

Another constraint in sunflower production is broomrape (*Orobanche cumana*), a parasitic flowering plant, that can cause significant yield loss of up to 100%. Most of the genes that confer resistance to broomrape were found to be monogenic dominant for broomrape races A to E and G (Vranceanu et al., 1980; Velasco et al., 2012), while resistance to race F was either inherited by a monogenic dominant gene (Pacureanu-Joita et al., 1998; Pérez-Vich et al., 2004) or by two recessive genes (Rodríguez-Ojeda et al., 2001) depending on the genetic background. Broomrape resistance genes are denoted as *Or* genes. Imerovski et al. (2014a) reported a single recessive resistance gene in the sunflower line HA-267 that carried a resistance gene higher than *Or_6_*. The majority of molecular analyses were conducted in investigating and creating different types of molecular markers for detection of *Or_5_* that conveys resistance to broomrape race E or lower (Lu et al., 2000; Tang et al., 2003a) (**Table 1**). The efficiency of RAPD and SSR primers in MAS for *Or_5_* were tested by Iuoras et al. (2004), however, none of the primers proved to be efficient or accurate enough. Imerovski et al. (2013) identified SSR markers associated with *Or_2_, Or_4_*, and *Or_6_* genes that could be used in converting broomrape susceptible sunflower genotypes into resistant ones. However, *O. cumana* populations belonging to race F have shown different aggressiveness (Molinero-Ruiz et al., 2009). Imerovski et al. (2016) mapped newly identified broomrape resistant gene conferring resistance to broomrape races overcoming race F from sunflower inbred line AB-VL-8 on LG3. The authors named the gene *Or_ab_*_-_*_vl_*_-8_, which was shown to be recessive and ORS683 mapped 1.5 cM from the gene. Further molecular analysis are needed in order to develop co-segregating markers for some of the *Or* genes. In addition, finding novel resistance sources is essential since broomrape races are emerging at a high speed. Recent work of Louarn et al. (2016) involved using 586, 985 SNPs from SUNRISE project^8^ on GeneTitan^®^ (Affymetrix) for identification of QTL for resistance to broomrape races F and G. The authors identified 17 QTL spread throughout 9 LGs. Among them was a stable QTL on LG13 that controlled the number of broomrape emergence that explained 15–30% of the phenotypic variability. This QTL was marked as the one that could be the most rapidly used. A molecular characterization of *O. cumana* populations in Europe using RAPD-PCR identified four groups (Molinero-Ruiz et al., 2014). These markers might be useful as molecular tools to detect first broomrape appearances in fields that had been free of virulent races (Molinero-Ruiz et al., 2014).

### Herbicide Tolerance

Different tolerances against herbicides inhibiting the large, catalytic subunit of the acetohydroxyacid synthase (AHASL) have become a very necessary tool in sunflower hybrid production and cultivation as these facilitate the application of either imidazolinones (IMIs) or sulfonylureas (SUs) against broadleaf weeds (Sala et al., 2012). It also allows a race independent control of broomrape (Skoric and Pacureanu, 2010). Three *AHASL* genes were isolated from sunflower: *AHASL1* located on LG9, *AHASL2* on LG6 and *AHASL3* on LG2 (Kolkman et al., 2004). Only mutations in *AHASL1* seem to be involved in the herbicide tolerance in sunflower. Four different mutated alleles have been explored for commercial use in sunflower hybrid breeding: Imisun/Clearfield^®^, Clearfield Plus^®^, Sures and ExpressSun^®^ (Sala et al., 2012). Point mutations from C-T in codon 205 (*Ahasl1-1*) and in codon 197 (*Ahasl1-2*) (adopting the *Arabidopsis* nomenclature) confer moderate tolerance to IMIs and high tolerance to SUs, respectively. The allele *Ahasl1-3* is characterized by a G-A mutation in codon 122 and results in high levels of IMI tolerance (Sala et al., 2008b). The broadest range of herbicide tolerance is shown by allele *Ahasl1-4*, which has a G-T mutation in codon 574 (Sala and Bulos, 2012). A first SNP marker based on the C-T change in codon 205 proved to be very useful as it cosegregated with partially dominant herbicide tolerance for the Imisun/Clearfield^®^ system (Kolkman et al., 2004), even though an additional non-target gene is required for the tolerance (Bruniard and Miller, 2001; Miller and Al-Khatib, 2002). One SSR marker exploiting the differences in the (ACC) repeats present in the *AHASL* gene allows the differentiation between the wild type *Ahasl1* allele and alleles *Ahasl1-1* and *Ahasl1-2* (Sures and ExpressSun^®^) (Kolkman et al., 2004; Bulos et al., 2013b). A CAPS marker developed by Bulos et al. (2013b) uses the A-T exchange to detect the *Ahasl1-3 allele* (Clearfield Plus^®^) by digesting the PCR product with the restriction enzyme *Bmg*BI. The markers can now help to select for herbicide tolerance. Nevertheless, the development of efficient screening tests for herbicide tolerance is crucial (e.g., Breccia et al., 2011; Vega et al., 2012).

### Seed Oil Quality

Several oil properties have been characterized as quantitative traits, however, some traits such as oleic acid content (OAC) could, to a certain extent, be considered a semi-qualitative trait since OAC is dependent not only on the environment, but also on the genetic background of the receiver line (Ferfuia et al., 2015; Regitano Neto et al., 2016). A partial duplication of the FAD2-1 allele caused by chemical mutation leads to an increase in OAC by silencing the FAD2-1 gene encoding FAD2 (oleoyl-phosphatidylcholine desaturase) (Lacombe et al., 2002; Schuppert et al., 2006). This enzyme catalyzes the synthesis of linoleic acid from oleic acid and by silencing its activity oleic acid is accumulated. Soldatov (1976) created the Pervenets cultivar with elevated OAC, which has become the main source of elevated OAC in sunflower breeding programs worldwide due to the beneficial properties of high oleic sunflower oil (Allman-Farinelli et al., 2005; Vannozzi, 2006). Inheritance of the OAC trait has been a subject of numerous studies and different results were reported from a single dominant gene to several genes influencing OAC (Urie, 1984; Lacombe et al., 2004; Joksimovic et al., 2006; Bervillé, 2010; Ferfuia and Vannozzi, 2015; Premnath et al., 2016; Dimitrijevic et al., 2017). Gene/genes involved in inheritance of OAC have been denoted as *Ol* genes. Different markers were employed in mapping and detecting the mutation (*Ol* mutation) in sunflower. The two RAPD markers, F15-690 and AC10-765, were linked with 7.0 and 7.2 cM to *Ol_1_* gene, respectively (Dehmer and Friedt, 1998). Later on, the *Ol_1_*-FAD2-1 locus was placed onto LG14 (Pérez-Vich et al., 2002; Schuppert et al., 2006). One major QTL identified by Pérez-Vich et al. (2002) explained 84.5% of the variation in the OAC. Schuppert et al. (2006) provided dominant INDEL markers for tracking the *Ol* mutation in addition to identifying 49 SNPs and five INDELs in the 3′-region of FAD2-1. Three years later, a co-dominant SSR marker tightly linked to the *Ol* mutation and dominant markers specific for the mutation were published (Lacombe et al., 2009). Recently, Premnath et al. (2016) identified in addition to the QTL on LG14, two additional QTL for OAC on LG8 and LG9. The two markers HO_Fsp_b for the QTL on LG14 (Schuppert et al., 2006) and ORS762 for the QTL on LG8 explained about 60% of the phenotypic variation in OAC. Several of the markers have been used for validation across numerous sunflower lines (Nagarathna et al., 2011; Singchai et al., 2013; Bilgen, 2016; Dimitrijevic et al., 2016). Dimitrijevic et al. (2017) reported marker F4-R1 created by Schuppert et al. (2006) as the most efficient in MAS for OAC.

### Fertility Restoration

Development of reliable tools for detection of cytoplasmic male sterility (*cms*) and restorer of fertility (*Rf*) genes would significantly improve and accelerate the process of developing sunflower hybrids. In sunflower, CMS PET1 originating from an interspecific hybridization of *H. petiolaris* with *H. annuus* (Leclercq, 1969) is the only CMS cytoplasm worldwide used for hybrid breeding. Male sterility is caused by the co-transcription of the *atpA* gene with the new CMS-specific *orfH522* leading to the expression of a 16-kDa-protein (Horn et al., 1991; Köhler et al., 1991). Fertility restoration suppresses the co-transcription anther-specific (Monéger et al., 1994). In sunflower, the restorer genes for the PET1 cytoplasm represent the best characterized due to the commercial use of this cytoplasm in sunflower hybrid breeding. The restorer gene *Rf_1_*, which was originally discovered by Kinman (1970) in the line T66006-2-1-B, has since then been integrated into a number of USDA/ARS RHA lines like RHA 271, RHA 272, RHA 273, and others (Korell et al., 1992; Serieys, 2005). A second major dominant restorer gene *Rf_2_* was discovered in a test cross between T66006-2-1-B and MZ01398. However, this *Rf_2_* gene seems to be ubiquitously present in almost all cultivated sunflower lines, along with maintainer lines of CMS PET1 (Serieys, 2005). Only *Rf_1_* is responsible for restoring male fertility in sunflower hybrids (Leclercq, 1984). RAPD markers in combination with AFLP markers were very useful for mapping of the restorer gene *Rf_1_* (Horn et al., 2003), which was first positioned on LG6 of the RFLP sunflower map (Gentzbittel et al., 1995). Two RAPD markers OPK13_454 and OPY10_740, which mapped 0.8 and 2.0 cM from *Rf_1_*, respectively, were converted into more reliable, easier to handle SCAR markers HRG01 and HRG02 (Horn et al., 2003). A recent study of these SCAR markers for breeding practice proved that HRG01 is more efficient for *Rf_1_* detection in perennial species, whereas HRG02 gave better results for annual species (Markin et al., 2017). In addition a multiplex TaqMan assay was established that allowed the detection of HRG01 and *orfH522* at the same time (Markin et al., 2017). Using the SSR markers ORS1030, *Rf_1_* had been mapped to LG13 (Kusterer et al., 2005) of the sunflower reference map (Tang et al., 2003b). In addition, a CAPS marker H13, which mapped 7.7 cM from *Rf_1_* gene, was developed from the RAPD marker OPH13_337 by digesting the PCR product with *Hinf*I (Kusterer et al., 2005). The tight linkage between CAPS H13 and *Rf_1_* was confirmed in *Xenia* hybrid combination (Port et al., 2013). An additional SSR marker ORS511 and a TRAP marker K11F05Sa12-160 were mapped to the *Rf_1_* gene with distances of 3.7 and 0.4 cM, respectively (Yue et al., 2010). A fertility restorer gene *Rf_3_*, which could be shown to be different from *Rf_1_* and *Rf_2_*, was identified in the confectionery restorer line RHA 280 (Jan and Vick, 2007). *Rf_3_* could be linked with eight markers to LG7, including five known SSR markers (ORS328, ORS331, ORS928, ORS966, and ORS1092) and three new SSR markers HT-619-1, HT619-2, and HT1013 derived from expressed sequence tags (Liu et al., 2012b). SSR ORS328, which mapped 0.7 cM distant from *Rf_3_*, represents so far the closest co-dominant marker to the gene (Liu et al., 2012b). Another restorer gene *Rf_3_* in RHA 340 has also been mapped to LG7 (Abratti et al., 2008). Rf ANN-1742, a restorer line derived from wild *H. annuus* showed resistance to rust (Qi et al., 2012b). The new rust resistance gene *R_11_* mapped with 1.6 cM closely to a restorer gene on the lower end of LG13. The SSR marker ORS728 was mapped 1.3 cM proximal from this restorer gene and 0.3 cM distal to *R_11_*. Marker analyses using HRG01, HRG02, STS115, and ORS728 indicated that this restorer gene, now called *Rf_5_*, might not be allelic to *Rf_1_* (Qi et al., 2012b).

So far, 72 new CMS sources have been described for sunflower (Serieys, 2005). However, only for very few of these CMS sources markers have been detected linked to the corresponding restorer genes (Horn et al., 2016). Feng and Jan (2008) tagged an additional restorer gene *Rf_4_* with molecular markers and assigned it to LG3 of the sunflower general reference map (Tang et al., 2003b). *Rf_4_* is restoring male fertility to a newly identified CMS cytoplasm GIG2. Schnabel et al. (2008) identified AFLP markers that mapped in close vicinity of the restorer gene *Rf_PEF1*, which represent a major restorer gene for the PEF1 CMS cytoplasm, another potentially interesting CMS source for commercial sunflower hybrid breeding. In addition, markers were developed that allowed the distinction between the PET1 cytoplasm and the PEF1 cytoplasm. For CMS 514A, a *H. tuberosus* based male sterile cytoplasm, the restorer gene *Rf_6_* was located on LG3 with eight markers (Liu et al., 2013). Two SSR markers, ORS13 and ORS1114, mapped as close as 1.6 cM to *Rf_6_*. GISH showed *Rf_6_* to be present on a small translocation introgressed from *H. angustifolius*.

Further analyses are needed in order to develop more tightly linked molecular markers to *Rf* genes to locate them on the genetic map and to get an insight on the fertility restoration mechanisms in sunflower. In other species, most of the so far cloned restorer of fertility genes belong to the pentatricopeptide repeat gene family (PPR), however, also other types of restorer genes have been identified (Horn et al., 2014).

## Association Mapping

For association mapping two approaches have been explored: (1) genome-wide association studies (GWAS) and (2) candidate gene approaches. For most plant species, the last strategy was predominantly applied because whole genome sequences have only recently become available (Fusari et al., 2008). However, high-throughput marker systems nowadays give full genome coverage, which makes approaches as genome-wide association mapping, QTLSeq mapping and genomic selection possible (Mammadov et al., 2012). As the linkage disequilibrium (LD) in sunflower rapidly decays (Liu and Burke, 2006; Kolkman et al., 2007; Fusari et al., 2008) studies based on associations could result in resolution levels detecting genes underlying quantitative trait loci. However, it is important to analyze the population structure of the association mapping population to avoid false associations.

In sunflower, only one of the association mapping studies so far was performed genome-wide (Mandel et al., 2013), all others were candidate gene based (Fusari et al., 2012; Cadic et al., 2013; Talukder et al., 2014b; Nambeesan et al., 2015; McAssey et al., 2016). Genome-wide association mapping was performed in an association population of 271 lines (Mandel et al., 2011), using 5,359 SNP marker from the Illumina Infinium Beadchip (Mandel et al., 2013). Associations were studied regarding flowering time, branching and heterotic groups. LD showed considerable variability across the genome, but significant marker-trait associations were detected. Selection for disease resistance as well as initial domestication might be responsible for the genome-wide differences in the LD profile (Mandel et al., 2013). This first screen was followed by a more detailed, refined association mapping approach based on candidate genes for branching (Nambeesan et al., 2015). Shoot branching was differentiated in no branching, apical, mid-apical, mid, mid-basal, basal branching as well as whole plant branching or other phenotype. A total of 48 candidate genes described to be involved in branching in other plant species were used to detect homologs to 39 genes in sunflower. Up to eight of the highest BLAST hit for each gene were included in the analyses due to the recent triplication of the sunflower genome (Badouin et al., 2017). For 13 candidate genes for branching co-localization of SNPs associated with branching was observed (Nambeesan et al., 2015). Most of these were found on LG10, where previous QTL mapping had detected the B-Locus for recessive branching (Tang et al., 2006; Bachlava et al., 2009). With regard to flowering time, a SNP in *HaFT2* was identified that co-localized with a flowering time QTL (McAssey et al., 2016).

Association mapping and linkage mapping were combined with QTL detection to identify mutations responsible for changes in flowering time (Cadic et al., 2013). Associations with flowering time could be demonstrated for 11 regions distributed over 10 LGs. In addition, QTL for flowering time were detected on 11 LGs in a RIL population by linkage mapping. This large number of QTL is consistent with the polygenic pattern of inheritance of flowering time reported before (Leon et al., 2000). SNPs detected by association mapping were then investigated with regard to positional overlaps with QTL identified in the RIL population. The remaining eight regions contained five candidate genes potentially associated with flowering time in other species that showed SNPs in sunflower, one of the genes was the gibberellin receptor GID1B (Cadic et al., 2013). Thirty genes, including this gene had before been investigated as candidate genes for flowering time with regard to domestication and improvement in sunflower (Blackman et al., 2011). One major QTL, which was detected on LG14 by linkage mapping (Poormohammad Kiani et al., 2009), was not detected by the association study (Cadic et al., 2013). This can happen if alleles are present in a low frequency in an association panel as one disadvantage of association mapping is that rare alleles are difficult to be associated with traits.

*Sclerotinia sclerotiorum*, a necrotrophic, fungal pathogen, is one of the most devastating diseases in sunflower. The fungus can cause three different types of diseases depending on which part of the plants gets infected and whether the infection occurs via ascospores or mycelia (Gulya et al., 1997). These are stalk rot, mid-stalk rot and head rot. In a panel consisting of 94 sunflower lines 16 candidate genes were screened for associations to *Sclerotinia* head rot using a Mixed Linear Model (MLM) that also considers family relationship as well as population structure (Fusari et al., 2012). These candidate genes had been derived from previous transcript profiling in sunflower (Peluffo, 2010) and *Brassica* (Zhao et al., 2007) after infecting the plants with *S. sclerotiorum*. Significant association of the haplotype 3 of the gene *HaRIC_B*, representing a truncated gene, was detected and accounted for 20% reduction in *Sclerotinia* head rot. Candidate gene association mapping for *Sclerotinia* stalk rot was also performed in another association panel of 260 cultivated sunflower lines (Talukder et al., 2014b). Eight genes, which had been identified in defense response against *S. sclerotiorum* in *Arabidopsis* (Guimaraes and Stotz, 2004; Guo and Stotz, 2007), served as basis to identify the orthologous genes in sunflower. The panel was divided in two groups representing either the best resistance response or the most susceptible lines. Association studies found strong association of *HaCOI1-1* and *HaCOI1-2* with resistance against *Sclerotinia* stalk rot, explaining 7.4% of the observed phenotypic variation (Talukder et al., 2014b).

Association mapping studies in the recent years have shown that this approach represents an interesting alternative to linkage mapping especially regarding quantitative inherited traits.

## Toward Genomic Selection

Genomic selection (GS) is so far mostly used in animals, e.g., dairy cattle (Van Raden et al., 2009). However, application of genomic selection got started as well in plant breeding, e.g., in maize (Massman et al., 2013; Bandeira e Sousa et al., 2017; Cantelmo et al., 2017; Lyra et al., 2017), potato (Habyarimana et al., 2017), soybean (de Azevedo Peixoto et al., 2017), sugar beet (Würschum et al., 2013), and wheat (Bassi et al., 2016). Genomic selection was regarded as promising in hybrid breeding of self-pollinating crops as wheat (Longin and Reif, 2014; Zhao et al., 2015), especially if little is known about the heterotic pools. To implement GS into sunflower breeding programs some general aspects of genomic selection need to be emphasized.

Genomic selection selects the individuals based on genomic breeding values (GEBVs) (Meuwissen et al., 2001). The idea of GS is to use genome-wide molecular data to effectively select for quantitative trait loci (Bernardo, 2008; Massman et al., 2013; Würschum et al., 2013). More than 10,000 QTL have been detected by traditional mapping approaches considering 12 major crop species, but only very few have been successfully applied in marker-assisted breeding programs (Bernardo, 2008). Genomic selection is a concept that becomes more attractive as high-throughput genotyping becomes feasible due to recent advances in genotyping platforms and to considerable price reductions in the last few years. As first step in GS, a training population has to be established that is genotyped and phenotyped. This training population is needed to adjust the statistical models, which are then applied to predict breeding and genotypic values of individuals that have not been phenotyped (Bassi et al., 2016). The breeding population consists of these not phenotyped individuals that are only genotyped. Selection is performed in the breeding population. Finally, a validation population serves to estimate the accuracy of the GS models (Bassi et al., 2016). Comparing traditional MAS and GS, three major differences are obvious: (1) within the training phase markers linked with a gene of interest and quantitative traits are identified in MAS, whereas in GS models are developed to predict GEBVs, (2) in the breeding phase only few markers are used in traditional MAS for genotyping, whereas in the GS genome-wide genome data are collected and (3) regarding the selection in the breeding phase traditional MAS uses only the identified markers to select the individuals by genotype, whereas selection in GS is performed based on the GEBV (Nakaya and Isobe, 2012). For the success of GS, the accuracy of the prediction of GEBV is the most important factor. The accuracy of prediction relies on the characteristics of the training population as size, marker density, trait heritability and kinship between training and breeding population as well as the ratio of training population : breeding population (Nakaya and Isobe, 2012; Bassi et al., 2016). In traditional MAS, markers tightly linked to a QTL could be applied in most other breeding population, so that the relationship between the mapping and the breeding population had not to be considered by the breeder. However, in GS the interrelationship between training and breeding population is crucial for the predictive power (Nakaya and Isobe, 2012).

In sunflower, prediction of hybrid performance was based on fingerprinting data in form of 572 AFLP markers (Reif et al., 2013). Intragroup (133) and intergroup hybrids and the parental lines were evaluated at two locations in 2 years for grain yield, oil content and oil yield. If no information on the General Combining Ability (GCA) of the parental lines was accessible, prediction of hybrid performance using genomic selection methods was accurate if the parents were closely related, but with genetically distant lines prediction proved challenging (Reif et al., 2013). However, prediction based on GCA could not be improved by genomic selection. In the recent years, large sets of markers were generated in sunflower by genotyping-by-sequencing (Baute et al., 2016; Celik et al., 2016; Talukder et al., 2016; Ma et al., 2017; Qi et al., 2017), application of the new 25 K SNP genotyping array (Livaja et al., 2016) and sequencing of parental lines (Mangin et al., 2017a). However, so far only SNP array data were used for genomic prediction of *Sclerotinia* resistance (Livaja et al., 2016) and sequencing data for the genomic prediction of sunflower hybrid oil content (Bonnafous et al., 2016; Mangin et al., 2017a). In the latter case, an incomplete factorial design consisting of 36 CMS lines and 36 restorer lines was used to compare prediction accuracy of GS and classical GCA modeling in sunflower. Multi-environmental field trials were performed to characterize 452 sunflower hybrids of the panel with regard to hybrid performance in oil content, which represents a primarily additive trait with high heritability. In addition, all 72 parental lines were sequenced to obtain genome-wide SNP markers (Mangin et al., 2017a). Genomic predictions were then made for missing hybrids and hybrid combinations lacking information about at least one parental line. In conclusion, GS led to considerable improvement in breeding efficiency compared to the conventional GCA modeling if little is known about one or both parental lines (Mangin et al., 2017a). For *Sclerotinia* midstalk rot, the prediction ability of a genome-based best linear unbiased prediction (GBLUB) model was evaluated in a biparental population genotyped with the 25 K SNP array (Livaja et al., 2016). High predictive abilities were obtained for “stem lesion length” and lower predictive abilities for “leaf lesion length” and “speed of fungal growth,” which represent traits with lower heritabilities. These first experimental trials for genomic predictions, using and comparing the results of different models, have shown the potential and the limitations for genomic selection in sunflower.

## Future Perspectives

In this review the emphasis was given to plant genetic resources and molecular tools used to detect and exploit genetic diversity and to facilitate sunflower hybrid breeding. Traditional MAS has been successfully used to introduce monogenic traits into the breeding material, especially disease resistance as well as herbicide tolerance. Validation of identified molecular markers across different genotypes has also shown the limitation in markers to be used in different genetic backgrounds. However, sunflower researchers have put a lot of effort in the identification of markers linked to specific traits without gaining insight into the function of the involved genes, even though this would allow a better understanding of the metabolism and mechanisms behind traits. Breeding for complex polygenic traits is still challenging. With this regard, it is necessary to stress the importance of precise phenotypic evaluation, on which molecular biologists rely to correctly interpret the molecular and phenotypic data. High-throughput phenotyping as applied and tested in other crops would be also interesting for sunflower (Sankaran et al., 2015). There has been a first report on testing remote sensing on sunflower and maize in China with regard to future applications (Yu and Shang, 2017). In recent years, high throughput genotyping platforms, e.g., SNP arrays, GBS and whole genome sequencing have been established and successfully used in sunflower (Livaja et al., 2016; Qi L. et al., 2016; Talukder et al., 2016). GWAS (genome wide association study) and GS (genomic selection) using large amounts of markers across a wide range of genotypes provided by these techniques open up new possibilities to address complex traits in sunflower. However, GWAS is still expensive and unavailable for many researchers and breeders. Some initial steps have been made in order to create the most appropriate models for prediction of hybrid performance based on GWAS and GS data (Bonnafous et al., 2016; Mangin et al., 2017a), yet there is still a need for further improvement of prediction models, which mostly take additive effects into account, whereas for heterosis also dominance and epistasis play an important role. As in conventional breeding, species-specific strategies will have to be developed for GS taking into account reproduction system, generation time, genome structure, harvested organs and breeding purposes (Nakaya and Isobe, 2012). However, first empirical GS studies in plants showed the potential for GS also in plant breeding. It could be demonstrated that the correct choice of population allows successful performance of GS even with lower numbers of markers and reasonable sizes of populations (Nakaya and Isobe, 2012).

Access to the recently published sunflower genome sequence (Badouin et al., 2017) should allow researchers and breeders to make sunflower breeding more efficient in the coming years. However, exploring the sunflower genome on its own is not enough. Extensive transcriptomics, proteomics and metabolomics data are required as only the combination of all Omics data will enable us to get to the bottom of some important physiological and molecular mechanisms unique to sunflower. This is especially important for quantitative traits such as drought tolerance or biotic stress resistance (e.g., against *Sclerotinia, Phoma, Phomopsis*). First results in this direction have been published. Transcriptional profiling has been done with regard to disease reactions of resistant and sensitive genotypes to pathogens as *S. sclerotiorum* (Muellenborn et al., 2011), *Plasmopara halstedii* (Livaja et al., 2013) and *Verticillium dahliae* (Guo et al., 2017). Identification of the differentially expressed genes now allows a better understanding of the mechanisms behind pathogen attacks and plant reactions. This knowledge will be helpful with regard to developing resistant cultivars. Earlier metabolome data of head rot between genotypes with different reactions to *S. sclerotiorum* also gave an indication to 63 metabolites involved in the attack of the pathogen (Peluffo et al., 2010). To analyze the response of sunflower to drought transcriptome analyses of sunflower genotypes under water-limited conditions in comparison to well-water plants have been performed by RNASeq or microarray analyses (Liang et al., 2017; Moschen et al., 2017; Sarazin et al., 2017). Combination of the transcriptomic and metabolic data made the identification of drought relevant hubs for transcription possible (Moschen et al., 2017). Leaf senescence is a naturally occurring process, but the onset and progress of senescence plays a major role for yield. Integration of transcriptomic and metabolomics data identified metabolites and transcription factors as applicable biomarkers (Moschen et al., 2016a,b). To explore the potential of other species in the genus *Helianthus* for sunflower breeding, transcriptomics have also been performed to address populations of, e.g., perennial sunflowers as *H. maximiliani* (Kawakami et al., 2014) and *H. tuberosus* (Jung et al., 2014) as well as interspecific hybrids of annuals in the first generation (Rowe and Rieseberg, 2013). Proteomic analyses in sunflower have been performed with regard to drought stress (Castillejo et al., 2008; Fulda et al., 2011; Ghaffari et al., 2013, 2017), cold acclimation (Balbuena et al., 2011), response to metal-ion contamination (Garcia et al., 2006; Printz et al., 2013; Lopes Júnior et al., 2015), seed protein composition (De Sousa Barbosa et al., 2013), heterosis performance (Mohayeji et al., 2014), and resistance to *O. cumana* (Yang et al., 2017). In addition, the sunflower genome database represents a very valuable tool, which allows access to a wide range of transcriptome data, which have already been successfully used to address flowering time and oil metabolism (Badouin et al., 2017). However, further studies in sunflower are still needed in order to analyze in detail responses to different abiotic and biotic stress conditions and to prepare sunflower for future climatic challenges. Combining Omics data will allow system biology approaches to improve sunflower hybrids. Another aspect is the optimization of plant architecture to a more compact form, which would have an influence on photosynthesis, lodging, climatic adaptation and possible plant densities. This could also improve sunflower hybrid performance and increase yields per hectare by use of higher plant densities (Hall et al., 2010). Picheny et al. (2017) used the crop model SUNFLO to design sunflower ideotypes with optimized morphological and physiological traits for certain environments.

However, only a combined effort of the sunflower research community can make sunflower more competitive to other oil crops. The new high-throughput technologies combined with new genomic-based breeding strategies give us the opportunity, as never before, to understand and mine genetic variation and to use it for improvement of sunflower hybrids.

## Author Contributions

Both authors AD and RH have made an equal substantial, direct and intellectual contribution to writing the review and its revision, and approved it for publication. The table was prepared by AD, the figure by RH. The authors complied to the ethical standards.

## Conflict of Interest Statement

The authors declare that the research was conducted in the absence of any commercial or financial relationships that could be construed as a potential conflict of interest.

## Footnotes

1 http://www.nsseme.com/about/inc/oilcrops/wild.php

2 http://pgrc3.agr.gc.ca/order-ordre_e.html

3 https://gbis.ipk-gatersleben.de/GBIS_I/

4 https://www.ars-grin.gov/

5 http://wwwx.inia.es/coleccionescrf/PasaporteCRF.asp

6 http://www.sunflower.uga.edu/cmap/

7 https://www.heliagene.org/HanXRQ-SUNRISE/

8 https://cnrgv.toulouse.inra.fr/fr/Projets/Analyse-de-genome-pour-l-amelioration-des-plantes/SUNRISE-SUNflower-Resources-to-Improve-yield-Stability-in-a-changing-Environment
